# *Dientamoeba fragilis* DNA detection in *Enterobius vermicularis* eggs

**DOI:** 10.1111/2049-632X.12071

**Published:** 2013-08-12

**Authors:** Jessica Ögren, Olaf Dienus, Sture Löfgren, Peter Iveroth, Andreas Matussek

**Affiliations:** 1 Clinical Microbiology Laboratory Department of Laboratory Medicine Division of Medical Services, County Hospital RyhovJönköping, Sweden; 2 Department of Infectious Disease Control, County Hospital RyhovJönköping, Sweden

**Keywords:** *Entrobius vermicularis*, *Dientamoeba fragilis*, DNA, transmission

## Abstract

*Dientamoeba fragilis* is an intestinal protozoan suspected of causing gastrointestinal symptoms, and its mode of transmission is unknown, although first described almost a century ago. A hypothesis is that *Enterobius vermicularis* is a vector for *D. fragilis*, and recently, *D. fragilis* DNA was detected within surface-sterilized eggs of *E. vermicularis*. Using real-time PCR, we detected *D. fragilis* DNA in 18 (85%) of 21 samples of *E. vermicularis* eggs collected from patients harbouring *D. fragilis* in faeces. This finding supports the hypothesis that *E. vermicularis* may have an important role in the transmission of *D. fragilis*.

This paper describes a protocol to wash and surface-sterilize *E. vermicularis* eggs, with the aim of showing presence of both *E. vermicularis* and *D. fragilis* specific DNA within, and the results from 20 co-infected patients. The study has merit as a confirmatory study of the trials by Röser *et al*. (2013), and includes improvements of the protocol.

*Dientamoeba fragilis* is a protozoan parasite of the human large intestine. A pathogenic role for *D. fragilis* has been suggested (Wenrich *et al*., 1935; Sapero, 1939; Girginkardesler *et al*., 2003; Johnson *et al*., 2004; Stark *et al*., 2010; Barratt *et al*., 2011a); however, there is lack of conclusive evidence of its virulence and the mechanisms involved. Although first described almost a century ago by Jepps & Dobell (1918), little is known about *D. fragilis* transmission modes (Barratt *et al*., 2011b). More than 50 years ago, Burrows & Swerdlow (1956) suggested that *Enterobius vermicularis* may serve as a vector for *D. fragilis*, and epidemiological data indicate a higher than expected co-incidence of *E. vermicularis* and *D. fragilis* in clinical samples (Girginkardesler *et al*., 2008). Recently, *D. fragilis*-specific DNA sequences could be amplified from DNA extracted from surface-sterilized *E. vermicularis* eggs (Röser *et al*., 2013). Our results indicate the presence of *D. fragilis* DNA in *E. vermicularis* eggs collected from patients harbouring *D. fragilis*.

Microscopic detection of *D. fragilis* was performed on faecal samples transported in sodium acetate formaldehyde (7 mL, 1.5%). Clear sticky tape (*n* = 80) and anal swab (in saline, *n* = 4) samples from children (*n* = 84) with *D. fragilis* in faeces were collected, and *E. vermicularis* eggs were detected in 21 of 84 (25%) of these samples by light microscopy.

*Enterobius vermicularis* eggs were detached by incubation of the tape in 1 mL ethyl acetate for 1 h in a head-over shaker. The solvent was transferred into a 1.5-mL reaction tube followed by centrifugation at 10 000 ***g*** for 2 min. Supernatants were discharged, and the pellets were then washed twice in PBS (pH 7.4) by centrifugation at 10 000 ***g*** for 2 min. *Enterobius vermicularis* eggs from swab samples were centrifuged at 10 000 ***g*** for 2 min, and pellets were then treated twice with a hypochlorite solution (0.5%) for 5 min. Finally, the hypochlorite-treated eggs were washed once in PBS (pH 7.4) as described above.

All pellets and the final wash solutions were then treated with 50 μL G2 buffer and 10 μL proteinase K (Qiagen, Hilden, Germany) at 56 °C for 1 h. Thereafter, 150 μL AL buffer was added followed by incubation for another 15 min at 56 °C. Tubes were then frozen at −180 °C for 15 min and finally heated at 98 °C for 15 min.

DNA was extracted and purified using the MagAttract DNA Mini M48 kit (Qiagen) in a M48 instrument (Qiagen), according to the manufacturer's instructions. DNA extracts were stored at −20 °C prior to PCR analysis. The presence of *D. fragilis-* and *E. vermicularis-*specific sequences were detected by a duplex real-time PCR on DNA extracted from the final wash solutions and *E. vermicularis* eggs in a LightCycler 480 II instrument (Roche Diagnostics GmbH, Mannheim, Germany). The detection of *D. fragilis* was performed using published primers (Verweij *et al*., 2007) and a modified TaqMan probe DientamoebaTM: LC670-AAGCAATTCTAGCCGCTTATCACATTATGCA-BBQ.

We designed the following novel primers and probe for *E. vermicularis* detection based on the 5S rRNA gene-IGS region using the online primer3 software (Primer-BLAST NCBI): E.vermicu F: 5′-ACAACACTTgCACgTCTCTTC, E.vermicu R: 5′-TAATTTCTCgTTCCggCTCA and probe E.vermicu TM: 6FAM-CCAAgCCACAgACTCACTgATgTTCA-BBQ (TIB MOLBIOL, Berlin, Germany).

The reaction mixture for the duplex real-time PCR contained 12.5 μL Roche LightCycler 480 Probes Master, 6 pmol of each primer, 4 pmol E.vermicu TM probe and 5 pmol DientamoebaTM probe and 5 μL template DNA in a total volume of 25 μL. The reaction conditions were 5 min at 95 °C, thereafter 50 cycles of 5 s at 95 °C and 15 s at 60 °C.

In total, 18 (85%) of 21 pellets contained *D. fragilis* sequences with cycle threshold (C_t_) values ranging from 29 to 43. All 21 samples also contained *E. vermicularis* sequences with C_t_ values ranging from 17 to 32. In two of 21 wash solutions, *D. fragilis* sequences were detected. The C_t_ values in the wash solution were, however, substantially higher than in the corresponding pellets (41 compared with 32 and 45 compared with 36). No *D. fragilis* sequences were detected in wash solutions from the hypochlorite-treated eggs (*n* = 4).

Our study indicates that eggs of *E. vermicularis* from a high proportion of patients co-infected with *D. fragilis* contain *D. fragilis* DNA, extending recent results published by Röser *et al*. (Röser *et al*., 2013). Culture of *D. fragilis* from the eggs would confirm viability, a prerequisite for transmission.

We detected low levels of *D. fragilis* DNA in two of 21 wash solutions indicating a minimal risk of possible DNA contamination. The detection of these low levels of *D. fragilis* DNA in the wash solutions may be explained by damage of *E. vermicularis* eggs in the last wash step. We are convinced that detected *D. fragilis* DNA originates from inside of *E. vermicularis* eggs.

In conclusion, we here detect *D. fragilis* DNA within the majority of extensively washed as well as surface-sterilized *E. vermicularis* eggs from patients with *D. fragilis*. This study indicates a possible and an important role of *E. vermicularis* in *D. fragilis* transmission, which may have implications for public health measures as well as therapeutic interventions.
